# Cocoa Flavanols: Natural Agents with Attenuating Effects on Metabolic Syndrome Risk Factors

**DOI:** 10.3390/nu11040751

**Published:** 2019-03-30

**Authors:** Maria Eugenia Jaramillo Flores

**Affiliations:** Departamento de Ingeniería Bioquímica, Escuela Nacional de Ciencias Biológicas-Instituto Politecnico Nacional, Wilfrido Massieu s/n esq, Manuel Stampa, Unidad Profesional Adolfo López Mateos, Alcaldía G. A. Madero, Ciudad de México CP 07738, Mexico

**Keywords:** cocoa, bioactive compounds, flavanols bioavailability, anti-inflammatory properties, metabolic syndrome, oxidative stress

## Abstract

The interest in cacao flavanols is still growing, as bioactive compounds with potential benefits in the prevention of chronic diseases associated with inflammation, oxidative stress and metabolic disorders. Several analytical methodologies support that the flavanols in cacao-derived products can be absorbed, have bioactive properties, and thus can be responsible for their beneficial effects on human health. However, it must be considered that their biological actions and underlying molecular mechanisms will depend on the concentrations achieved in their target tissues. Based on the antioxidant properties of cacao flavanols, this review focuses on recent advances in research regarding their potential to improve metabolic syndrome risk factors. Additionally, it has included other secondary plant metabolites that have been investigated for their protective effects against metabolic syndrome. Studies using laboratory animals or human subjects represent strong available evidence for biological effects of cacao flavanols. Nevertheless, in vitro studies are also included to provide an overview of these phytochemical mechanisms of action. Further studies are needed to determine if the main cacao flavanols or their metabolites are responsible for the observed health benefits and which are their precise molecular mechanisms.

## 1. Introduction

The three main cultivars of cacao beans are: Criollo, Forastero and Trinitario. However, with the aim of increasing production and resistance to pests, new varieties have been created from the three original cultivars. Forastero is the most widespread (mainly in Africa) and used around the world, due to its high adaptability and resistance to pests. Criollo is native from Mexico, Central and South America. It is considered the most ancient cultivar and is appreciated for its high quality, flavor and aroma. However, its production represents only 5% of the world cacao beans production, due to its low resistance to pests. Trinitario is a hybrid between the Criollo and Forastero trees that combines good-quality flavor and aroma with pest and disease resistance [1,2].

The cacao bean is a fruit widely recognized as one of the main sources of phenolic compounds with the highest flavanols content of all foods on a per weight basis [3]. The content and profile of bioactive compounds of the cocoa depends on a number of factors such as type and quality of the crop, place of culture, type of process (fermentation, drying and roasting), such that in 6 samples of cocoa liquor around the world, the highest to least quantity of the following components: (-)-epicatechin is presented in samples from Ghana, followed by Mexico and Venezuela; (+)-catechin from Sao Tome and Ghana; caffeic acid derivatives from Venezuela, Ecuador and Ghana; (-)-gallocatechin (GC) from Ecuador, Madagascar and Mexico, without presence in the rest of the samples; (-) - epigallocatechin (EGC) from Madagascar, Ecuador and Sao Tome without the presence of these compounds in Mexico and Venezuela; caffeine from Ecuador, Venezuela and Mexico; and theobromine from Sao Tome, Ghana and Madagascar [4]. About 13 flavanols have been detected and quantified, mainly (-)–epicatechin (0.12–2.83 mg/g), (+)–catechin (0.040-0.090 mg/g), epigallocatechin, epigallocatechin-3-gallate and procyanidins B1(0.035 mg/g), B2 with three different isomers (0.13–0.97 mg/g), B3, B4 with 2 isomers, C1 and D; 7 flavones—luteolin orientin, isoorientin, apigenin, vitexin, ixovitexin; 4 flavanones—naringenin, prunin, hesperidin, eriodyctyol; 4 flavonols—quercetin (0.21–3.25 µg/g), quercetin 3-o-arabinoside (2.1–3.2 µg/g), isoquercitrin (4–4.3 µg/g), and hyperoside; 4 anthocyanidins—cyanidin, and 3 different glycosylated cyanidins; and finally, 8 fenolic acids—vanillic acid, syringic acid, chlorogenic acid, phlorectic acid, coumaric acid, caffeic acid, ferulic acid and phenilacetic acid [3].

Epidemiological and clinical studies show and confirm that regular intake of cocoa powder and/or dark chocolate (50%–70% cacao) is related to a decrease in systolic blood pressure (SBP) (−3.2 to −5.88 mmHg) and diastolic blood pressure (DBP) (−2.0 to −3.30 mmHg), as well as to an improvement of the vascular endothelial function (measured as a function of endothelium vasodilatation and an increase in the production of nitric oxide) in groups with some type of cardiovascular disease or with multiple risk factors [5,6,7]. Cocoa flavanols reduce the blood pressure by increasing the availability of nitric oxide (increasing the nitric oxide synthase activity and reducing the oxidative stress), consequently vasodilatation increases and finally blood pressure is reduced; or, by inhibition of angiotensin-converting enzyme, interrupting the chain of reactions of angiotensinogen that by the action of renin produces angiotensin I which in turn by action of angiotensin converting enzyme (ACE) is transformed into angiotensin II and finally increases blood pressure [8].

Metabolic syndrome (MS) is a heterogeneous group of correlated metabolic disorders that occur together and raise the risk for diabetes type II (DM2) and cardiovascular diseases with high rates of morbi-mortality [9,10,11]. To diagnose MS, the International Diabetes Federation (IDF) considers the presence of abdominal obesity (defined as waist circumference with ethnicity specific values) as the main risk factor, plus two additional symptoms: i) high blood pressure (SBP: ≥10 mmHg; DBP: ≥85 mmHg or a specific treatment for hypertension arterial (HTA)); ii) high plasmatic triglycerides (≥150 mg/dL or a treatment specific for this disorder); iii) low HDL-cholesterol (M: <40 mg/dL; W: <50 mg/dL); iv) impaired fasting glycemia (≥100 mg/dL) or diabetes mellitus type 2 (DM2) diagnosis [12].

Several clinical and epidemiological studies have demonstrated that the intake of flavonoids found in vegetables, fruits and oilseeds reduce the risk of developing several types of non-communicable chronic diseases derived from metabolic disorders [13,14].

Among the vast group of flavonoids found in nature, flavanols are a sub-group of particular interest since it has shown multiple protective effects against diseases associated with metabolic and oxidative stress [15].

## 2. Cacao Flavanols

More than 200 chemical compounds have been identified in cacao beans, and most of them are stored in the vacuoles of the so-called “polyphenolic cells” [7]. The polyphenol content makes up about 12%–18% of the whole bean’s dry weight. Approximately 60% of the total polyphenols content in non-fermented cacao beans corresponds to monomeric (catechin and epicatechin) and oligomeric flavanols. The main monomeric flavanol is (−)-epicatechin (with up to 35% of polyphenol content), followed by (+)-catechin and procyanidin B2 (epicatechin-(4β-8)-epicatechin) [16]. Flavanols are secondary metabolites that belong to a sub-class of a larger group of plant compounds known as flavonoids. They share a general chemical structure that includes two rings (A and B) linked through three carbons that form an oxygenated heterocyclic ring (C). As a particular feature, flavanols have multiple hydroxyl groups on the A, B and C rings that have been associated with a decrease in oxidative stress markers. As with all bioactive substances, flavanols and procyanidins mechanisms are largely dependent on their bioavailability at their target tissue [17,18].

## 3. Flavanol Bioavailability

Upon ingestion, flavanols bioavailability depends on their absorption, metabolism at the gastrointestinal tract, tissue and cellular distribution, and tissue metabolism.

In vitro study showed that procyanidins are hydrolyzed into oligomers when passing through the gastric lumen, due to the high acidity of the medium (pH 2) [19]. Given the above, it was hypothesized that gastric de-polymerization of procyanidins favors their absorption by the small intestine. However, in vivo studies in both animals and humans have shown that both monomeric and oligomeric flavanols remain stable during gastric digestion [20,21,22,23]. While the gastric concentrations of epicatechin and procyanidins B2, B5 (dimeric) and C1 (trimeric) did not significantly change over the stomach transit period [19]. 

Depolymerization of procyanidins (composed mainly by epicatechin monomers) would have resulted in an increase in the epicatechin gastric concentrations and a change in the ratios of oligomers to epicathechin and catechin to epicatechin. However, neither hydrolysis of procyanidins nor change in the aforementioned ratios were observed [20].

Once monomeric and oligomeric flavanols reach the small intestine, they can undergo a series of biotransformations (mainly of phase II) that produce O-methylated, O-sulfated, and O-glucuronidated metabolites, which can be absorbed into the blood stream. Procyanidins with a high degree of polymerization cannot be absorbed in the small intestine, so they reach the colon to be used for microbial catabolism, which leads to the formation of smaller phenolic compounds capable of reaching the liver and then undergo phase II conjugation [24,25]. This has been demonstrated in previous in vivo absorption kinetic studies, in which the presence of these metabolites was observed in the plasma (in concentrations of macro to nanomoles) 30 to 60 minutes after the ingestion of cocoa-based beverages [16,20,21]. The monomers epicatechin and catechin showed the highest absorption rates (22%–55%), while dimeric and trimeric procyanidins were less absorbed (equal or less than 0.5%) [16]. Using liquid chromatography-mass spectrometry, total concentrations of (-)-epicatechin, (+)-catechin and procyanidin B2 were quantified after 30 and 120 minutes after the intake of 0.375 g of cocoa/kg of body weight in healthy subjects (average intake of 26.4 g of cocoa: 323 mg monomers; 256 mg dimers). At both times, it was observed that the plasma epicatechin levels were higher than those of procyanidin B2 (0.5 h: 2.61 ± 0.46 μmol/L as compared to 16 ± 5 nmol/L and 2 h: 5.92 ± 0.60 μmol/L as compared to 41 ± 4 nmol/L, respectively) [26].

Table 1 shows the results of different studies on the bioavailability of pure flavanol compounds (catechin, epicatechin and procyanidin B2). As observed, the maximum plasma concentration is reached about 1 h after administration and is dose-dependent. Regarding (-)-epicatechin, their methylated and non-methylated metabolites were produced almost in the same proportion. On the other hand, catechin produces three more times non-methylated than methylated metabolites. When catechin and epicatechin are given as a mix, their metabolite profile remains similar to the one observed when both compounds are administered alone. After the oral administration of (+)-catechin, (−)-epicatechin and a mixture of the two, it can be shown that (-)-epicatechin is the flavanol with the highest absorption, even though (+)-catechin is also a monomer and its plasma metabolites are similar to the ones produced by (-)-epicatechin (glucuronides, sulfates and sulfoglucuronides) [27]. Similar results have been reported in bioavailability studies of epicatechin and procyanidin B2, when administered individually. The absorption of epicatechin was evaluated after oral administration of different doses of cocoa powder or the pure compound. Results showed that bioavailability of (-)-epicatechin present in cocoa powder was absorbed as efficiently as (-)-epicatechin administered alone [28].

A study in healthy volunteers analyzed the postprandial profile of (-)-epicatechin plasma metabolite profiles after oral consumption of a cocoa beverage. Through the combination of different enzymatic hydrolysis (arylsulfatase and β-glucuronidase) and the use of de novo chemically synthesized reference standards, it was possible to identify and measure 8 circulating postprandial (-)-epicatechin metabolites. As reported in previous studies, (-)-epicatechin-3′-β-D-glucuronide was the most abundant metabolite, followed by (-)-epicatechin-3′-sulfate and 3′-O-methyl-(−)-epicatechin-5/7-sulfate. It was also shown that O-sulfonation is a key conjugating reaction in the metabolism of (−)-epicatechin in humans, since the group of (−)-epicatechin sulfates resulted in being the most diverse [29].

After the intake of 100 g of dark chocolate (70% cacao), have been identified (-)-epicatechin metabolites in human plasma and urine [30]. In this study were identified 10 (-)-epicatechin metabolites, of which (-)-epicatechin-3′-β-D-glucuronide, (-)-epicatechin-3′-sulfate and 3′-O-methyl-(-)-epicatechin 5-sulfate were the major metabolites. Finally, (-)-epicatechin metabolite profile could be divided into three groups (glucuronides, sulfates, and O-methyl sulfates) and their distribution might be modified depending on the amount of (-)-epicatechin ingested, due to enzymatic activity [30].

**Figure 1:**
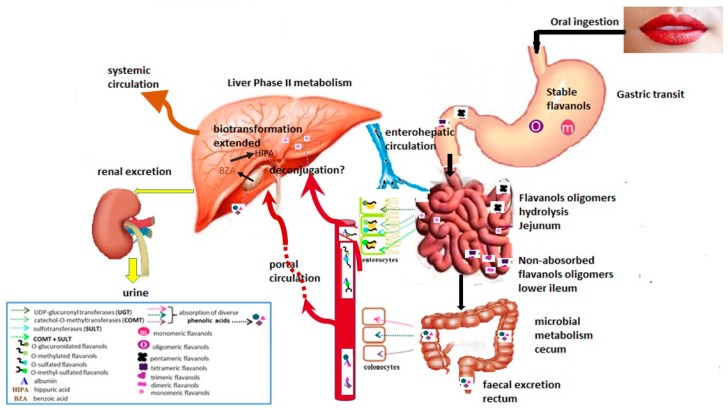
Cocoa flavanols bioavailability. Depolymerization of cocoa procyanidins in the stomach is negligible after ingestion of cacao-derived food products. Thus, most of them reach the small intestine unchanged. Once in the upper intestine, the flavanol monomers and oligomers undergo extensive metabolism (mainly phase II reactions: catechol-o-methytransferase (COMT), sulfotransferase (SULT) and uridine 5 diphosphate glucuronilsyltransferase (UGT)) within the enterocyte (jejunum) that gives rise to a range of O-methylated, O-glucuronidated, and O-sulfated flavanol derivatives. After absorption, the conjugated metabolites are bound to albumin and transported to the liver via the portal vein. Inside the hepatocytes, cocoa flavanols experience extended phase II biotransformations. The resulting metabolites can take 3 different pathways: reach other tissues through systemic circulation or get back to the duodenum through the bile (enterohepatic circulation) or be excreted in the urine. The fraction of the ingested cocoa procyanidins that are not absorbed in the small intestine can be metabolized by colonic microflora (lower part of the ileum and the cecum) into several phenolic acids (such as phenyl propionic acid, phenyl acetic acid and benzoic acid derivatives). These compounds may further be metabolized in the liver and undergo renal excretion, although some may enter other tissues.

Given the above, Figure 1 shows that in both humans and rats a large proportion of (-)-epicatechin (approximately 90%) is absorbed in different conjugated metabolites (glucuronides, sulfates, methylates) that are produced in the intestinal mucous lining. Through portal circulation, these compounds are transported to the liver where they undergo different biotransformation reactions of phase I and II, which produce new O-sulfated, O-glucuronidated and O-methylated forms of (-)-epicatechin. Afterwards, these new forms can be distributed among tissues or can be excreted from the body via bile or urine. The fraction of polymeric procyanidins unabsorbed in the upper gastrointestinal tract can reach the colon and become available for the local microbiota. Gut flora can produce several low–molecular-weight metabolites that can then be efficiently absorbed [16,20,21,31].

Regarding the absorption of procyanidin B2, it has been reported that it is less efficiently and less rapidly absorbed than epicatechin (5%–10% of the absorbed concentration of (-)-epicatechin), and, therefore, its human and rat plasma concentration is lower (10–40 nM/L) [27,32]. 

Using liquid chromatography and mass spectrometry, studies of kinetic absorption in humans and rats revealed that procyanidin B2 reachs a maximum plasma concentration at 30 to 60 min after administration of the pure compound [27]. Another study showed that human plasma level of procyanidin B2 reached the maximum at about 2 h after the consumption of a flavanol-rich cocoa [26]. 

However, a subsequent study showed that the maximal concentrations (*C*max) for total (^14^C) in blood were not attained until 5 to 6 h after oral administration (10.5 and 21 mg/kg) in rats [23]. Therefore, it was suggested that much of the radioactivity was absorbed from the distal part of the small intestine and/or the colon, whereas plasma concentrations of procyanidin B2 detected 30 min and 2 h after oral administration corresponded to the absorption in the proximal part of the small intestine.

Considering the low levels of procyanidin dimer B2 detected in human plasma and that little was known about its colonic metabolism, the catabolism by human faecal microbiota of (-)-epicatechin and procyanidin B2 was compared using an in vitro culture model. Results showed that from 10 phenolic acid catabolites common to both substrates, solely five phenolic catabolites were unique to procyanidin B2 [23]. Although full characterization and further investigation of these catabolites is needed, it has been suggested that they might be of interest with regard to potential biological effects.

Another topic of discussion regarding procyanidin B2 absorption is its possible biotransformations at the gastrointestinal tract. An in vitro study where human gastric conditions were simulated (gastric juice (pH 2.0) at 37 °C for up to 3.5), showed that oligomeric procyanidins (dimer to hexamer) decompose essentially to epicatechin monomeric and dimeric units. It was also suggested that the latter were the major components for absorption via the small intestine [19].

In a following study, the perfusion of isolated small intestine with cocoa procyanidin dimers B2 and B5 (50 mM) showed that both forms are transferred to the serosal side of enterocytes in a lesser extent than the monomer subunits (<1%). Instead, it was observed that unconjugated (-)-epicatechin was the most abundant bioavailable form of procyanidin B2 in plasma (95.8%). These observations provide an explanation for the high ratio of epicatechin to catechin observed [26]. In addition, the latter confirmed the presence of modest concentrations of procyanidin dimers (<1%) in human plasma after the intake of a flavanol-rich cocoa beverage [26]. Small amounts of methylated B2 dimer have also been detected in plasma (3.2%) after ex vivo perfusion of a rat small intestine [19].

Using an in situ rat small intestinal perfusion model it was shown that the presence of tetrameric procyanidins enhanced the absorption of procyanidin B2. These results are consistent with the findings, where elevated concentrations of procyanidin B2 was detected when fed in combination with high-degree of polymerization (DP) oligomers (>DP8) [32,33].

This suggests that further studies are needed to fully understand the synergy between procyanidins with different degrees of polymerization, particularly when considering that these coexist naturally in foodstuffs.

Hepatic glucuronidation, sulfation and methylation of procyanidin B2 have also been assessed using mice microsomal incubations. Unlike (-)-epicatechin, it has been shown that procyanidin B2 remains mostly unmetabolized. Only a small percentage was converted to four minor glucuronide products, although formation mechanisms have not yet been determined due to their low concentrations. This confirms that most of the biotransformations experienced by procyanidin B2 take place in the colon [22,23,34].

A wide range of phenolic acid microbial metabolites (high and low molecular weight) derived from (-)-epicatechin and procyanidin B2 biotransformations have been detected in urine samples collected after consumption of cocoa in humans and rats. It is noteworthy that the variations in the urinary excretion profiles in humans and rats may be influenced by the differences in the ingested dose of cocoa and to the different microbiota present in the intestine of each species [34].

After cocoa consumption, the major microbial metabolites found in human urine samples were caffeic acid, ferulic acid, 3-hydroxyphenylacetic acid, vanillic acid, 3-hydroxybenzoic acid, hippuric acid, 4-hydroxyhippuric acid, (-)-epicatechin and procyanidin B2. On the other hand, the major metabolites in rat urine samples were 3,4-dihydroxyphenylpropionic acid, cumaric acid, 3-hydroxyphenylacetic acid, protocatechuic acid, vanillic acid and (-)-epicatechin [34].

There are few studies regarding the colonic metabolism of phenolic compounds, and even fewer regarding oligomeric flavanols. However, it is now known that colonic microbiota has a large catalytic potential for enzymatic degradation of flavonoids, which results in a huge array of new metabolites with several biological and health-promoting properties [20,23,35,36].

## 4. Cacao Flavanols and Their Health Effects

After ingestion, flavanols can undergo significant modifications that result in several bioactive molecules with beneficial effects in chronic diseases related to metabolic disorders and oxidative stress. The mechanisms that have been proposed to explain the biological actions of flavanols are based on their capacity to act as antioxidants and to interact with signaling proteins, enzymes, DNA and membranes. According to the concentrations achieved in their target tissues, their mechanisms have been classified as direct (high concentration) or indirect (low concentration).

### 4.1. Direct Mechanisms

Until now, the most studied direct effects of cacao flavanols are related to their antioxidant capacity. It is well documented that the latter depends on their aromatic rings with hydroxyl substituents, which give flavanols an adequate configuration to act as electron donors (e^−^) and thus stabilize free radicals [18]. 

On the other hand, the degree of polymerization, partition coefficient and number and distribution of hydroxyl groups will influence the type of interactions that occur between flavanols and the cell membrane. For example, flavanols can partition in the hydrophobic core of membranes or form hydrogen bonds with the polar headgroups of membrane lipids [37].

Given the above, it has been proposed that flavanols may protect the integrity and function of the cell membrane by modulating changes in its fluidity and permeability produced by molecules with oxidation potential [37,38].

It is well known that when membrane fluidity decreases it is more prone to be oxidized. Instead, when fluidity increases membrane lipids are less exposed to oxidation. The effects of cocoa procyanidins on bilayer fluidity and susceptibility to oxidation have been studied using predominantly Jurkat T cells and liposomes. Cocoa derived dimers showed to protect Jurkat T cells from AMVN (2,2’-azobis (2,4-dimethylvaleronitrile)-mediated oxidation and to increase membrane fluidity, measured by a decrease in 1,6-diphenyl-1,3,5-hexatriene (DPH) fluorescence polarization. It was proposed that this effect could be mediated through complex interactions of dimers with membrane proteins, rather than lipids [37].

This was confirmed when the interaction of flavanols and procyanidins (dimers to hexamers) with liposomes (composed of phosphatidylcholine and phosphatidylserin) did not influence its membrane fluidity or lipid lateral phase separation [39].

Membrane lipid oxidation induces the formation of pores that allow the leakage of certain molecules. The increase in membrane permeability due to lipid oxidation has also been studied in liposomes oxidized with AMVN or ferrous iron. An in vitro study showed that preincubation with procyanidins significantly reduces the effect of ferrous iron on liposome permeability [37].

Taking into consideration flavanols’ interactions with cell membrane, lipid peroxidation has been widely used to study flavanols’ effects against oxidative stress. Breaking initiation and propagation reactions are considered as the most important antioxidant strategy of flavanols for inhibiting or retarding lipid oxidation. During initiation, free radicals (generally HO• and •O_2_^−^) substract a hydrogen (H) atom from membrane polyunsaturated fatty acids (PUFA) methylene groups [40]. The unpaired electron on the carbon is stabilized by a molecular rearrangement of the double bonds to form a conjugated diene, which then combines with oxygen to form a peroxyl radical (LOO•). The latter has the potential to extend the damage by reacting with other polyunsaturated fatty acids to produce lipoperoxides (LOOH) that can subtract hydrogen atoms from another polyunsaturated fatty acids (PUFA) (propagation reaction). This chain reaction generates irreversible structural and functional damages in cell membranes [18,40].

Additionally, there has been a growing interest in the ability of flavanols to chelate redox-active metals (iron and copper). In biological systems, oxidative stress breaks iron homeostasis and increases its intracellular concentration, promoting free radical-producing reactions and increasing DNA oxidative damage [41,42].

In the presence of hydrogen peroxide (H_2_O_2_)_,_ redox active metal ions such as Fe^2+^ or Cu^+^ that are covalently bound to the nucleotide bases of DNA react with it to form highly reactive hydroxyl radical (•OH). The latter abstracts a hydrogen atom from the deoxyribose sugar backbone, which in turn promotes the phosphodiester backbone cleavage and strand scission. Together, nucleotide bases damage (oxidation) and strand breakage have been associated to genetic mutations, cancer and cell death. Flavanols metal chelating properties reside in the presence of a catechol group (B ring) and hydroxyl substituents, since they are centers of high affinity for metal ions. However, there have been observed differences in the magnitude of their chelating activity depending on modifications in their chemical structure [41,42].

Mechanisms underlying flavanols direct effects have been mainly assessed by in vitro studies. However, in vivo studies (biological systems) have been useful for evaluating in vitro evidence.

The beneficial effect of (-)-epicatechin on lipid peroxidation was evaluated in ApoE knockout rats. Administration of epicatechin (64 mg/kg body weight) during 20 weeks significantly reduced aortic F2-isoprostanes, vascular superoxide and endothelin-1 production (*p* < 0.05 versus control ApoE(−/−) mice) [43,44].

Considering that direct effects require the presence of high concentrations of flavanols, it is thought that in living organisms these effects can be observed just in the gastrointestinal tract, since their absorption has shown to be limited [18].

Cocoa polyphenols are expected to activate Nrf2, which induces the transcription of antioxidant enzymes such as glutathione peroxidase, superoxide dismutase, and heme oxygenase 1, thus blocking the production of reactive oxygen species (ROS) and nitric oxide synthase (NOS), and attenuating oxidative stress, as well as a number of cellular kinases, including the mitogenactivated protein kinases (MAPKs) [45,46]. In Zucker diabetic fatty (ZDF) rats, the ingestion of a cocoa-rich diet (10%) for 9 weeks attenuated hyperglycemia, improved insulin sensitivity, and increased β-cell mass and function. At molecular level, cocoa intake prevented β-cell apoptosis by increasing antiapoptotic proteins (Bcl-xL) and decreasing proapoptotic proteins (Bax and caspase-3 activity) [47]. 

### 4.2. Indirect Mechanisms

A major limitation for the direct effects of flavanols in vivo conditions is their relatively low bioavailability. Therefore, it has been suggested that in living organisms the main effects of flavanols are mediated through modifications of enzymatic activities (induction or inhibition), receptors-ligand binding, regulation of protein synthesis and activities, transcription factors binding to their specific sites in DNA, among others (indirect mechanisms) [13,15,48]. In the particular case of the metabolic syndrome, indirect mechanisms have been the most studied. Both in vivo and in vitro models have been useful to link physiological mechanisms with health effects.

**Figure 2:**
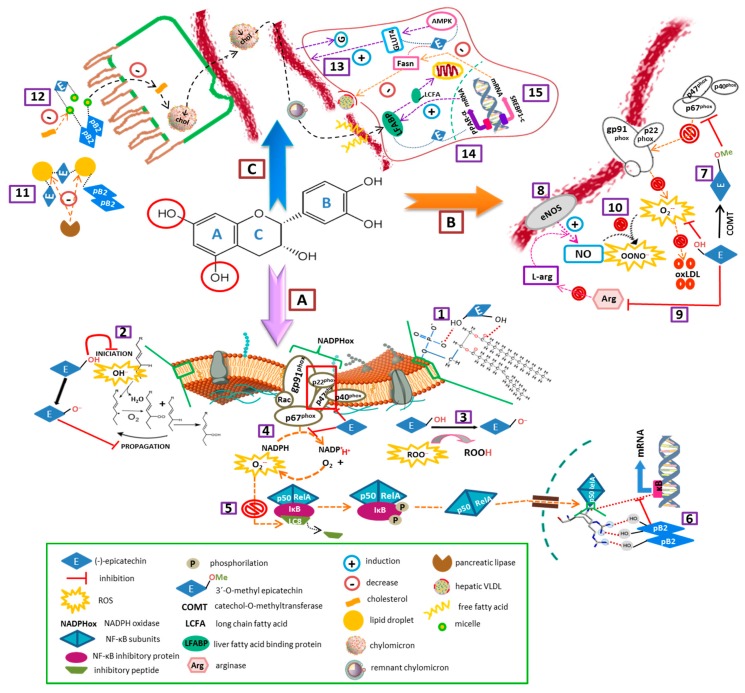
Mechanisms underlying the effects of cocoa (-)-epicatechin and procyanidin B2 on some of the major risk factors for developing metabolic syndrome. A and B mechanisms involved in flavanols antioxidant and anti-inflammatory effects. ① The presence of both hydrophobic and hydrophilic domains in flavanol molecules allow them to be adsorbed on the polar head of membrane lipids and/or to interact with the hydrophobic chains of lipids inside the bilayer in order to modify membrane fluidity and permeability. ② When inserted into the lipid bilayer, flavanols are in close proximity to scavenge free oxygen radicals (such as HO^−^) and lipid soluble radicals (L^−^, LOO^−^) derived from lipid peroxidation. ③ Scavenging free radicals is considered as one of the most important antioxidant mechanisms of flavanols, due to their OH groups (one-electron donation) and their aromatic structures (stabilization by resonance of the resultant radicals). ④ A high dose of epicatechin can prevent upregulation of nicotinamide dinucleotide phosphate NADPH oxidase subunits p47phox and p22phox, the increased enzyme complex activity and generation of O_2_^−^. ⑤ A decrease in cell reactive oxygen species (ROS) decreases the redox-sensitive release of the LC8 inhibitory peptide, preventing IκBα phosphorylation and degradation and the release of the active NF-κB complex. ⑥ Inside the nucleus, procyanidin B2 can mimic the guanine pairs in the κB DNA sequence and establish hydrogen bonds similar to those that specifically interact with the arginine residues of both p50 and RelA. This inhibits the interaction of NF-κB with κB sites in gene promoters and dependent gene transcription. ⑦ 3’-O-Methyl-(-)-epicatechin has shown to be an inhibitor of endothelial NADPH oxidase by blocking the translocation and interaction of p47phox/p67phox/p40phox with gp91phox and p22phox (transmembranal subunits). Thus, epicatechin improves bioavailability and bioactivity of NO in the arterial vascular endothelium. ⑧ Epicatechin can also increase circulating NO pool via eNOS activation. ⑨ (-)-epicatechin has shown to down-regulate endothelial cell arginase expression and activity. This leads to an increase in vascular L-arginine pool and substrate supply for the eNOS-catalyzed NO synthesis. ⑩ An adequate supply of L-arginine avoids eNOS uncoupling and formation of large quantities of O_2_^−^, which can scavenge NO to generate peroxynitrite or enhance the production of oxLDL. C. Mechanisms involved in flavanols hypolipidemic and hypoglycemic effects. ⑪ (-)-epicatechin and related flavanol oligomers may suppress triglyceride intestinal absorption by blocking the interaction between pancreatic lipase and the surface of emulsified lipid droplets. ⑫ Monomeric and oligomeric flavanols can lower plasma cholesterol concentrations by decreasing its solubility in intestinal micelles. ⑬ Cocoa flavanols could ameliorate hyperglycaemia by promoting translocation of GLUT4 in insulin-sensitive tissues via activation of AMPK signaling pathways. ⑭ Cocoa flavanols could also attenuate Non-alcoholic steatohepatitis (NASH) by increasing intracellular trafficking of (Long Chain Fatty Acid (LCFA) via liver fatty acid binding protein (LFABP) mRNA and protein expression. ⑮ In addition, cocoa flavanols may decrease fatty acid synthesis by down-regulation of the *Fasn* gene and protein expression.

Figure 2 summarizes the main mechanisms underlying the effects of cocoa (-)-epicatechin and procyanidin B2 on specific risk factors associated with the development of metabolic syndrome.

#### 4.2.1. Effects on Inflammation and Oxidation

Oxidative stress and inflammation are factors with great potential to exacerbate the progression of the metabolic syndrome. Visceral fat accumulation (central obesity) is closely related to an increase in ROS production and to the expression of inflammation-related genes (TNF-α, CRP, IL-6, IL-18, NF-κβ) that increase the risk of developing non-communicable chronic diseases [49,50].

Together, the antioxidant capacity of flavanols and the inverse association of cocoa and chocolate intake and the development of cardiovascular diseases have suggested that these compounds have the potential to reduce oxidative stress and inflammatory processes. Cocoa polyphenols diffuse into the cell and can inhibit MAPKs, thus blocking inflammatory transcription factors, such as NF-kB and AP-1. Together these signals repress the expression of inflammatory genes of many proinflammatory mediators, eg. TNF-α, IL-6, IL-8, IL-1, MCP-1, NO [45].

Studies in animal models have reported that supplementation of high-fat diets with cocoa decreases the plasma concentrations of inflammatory mediators (such as IL-6 and the monocyte chemoattractant protein-1 (MCP-1)), as well as the expression of genes encoding pro-inflammatory molecules (*IL-6, IL-12b* and *NOS 2*) in white adipose tissue [51,52].

##### NADPH Oxidase and Endotheline 1

Given the importance of nitric oxide (NO) on the regulation of vascular homeostasis, the enzymes and transcription factors involved in the decrease in NO synthesis and bioavailability have been proposed as targets for the action of cacao flavanols. Such is the case of NADPH oxidase (Nox), an enzymatic complex that catalyzes the formation of ROS (mainly O_2_^−^ and de H_2_O_2_), reducing the bioavailability of NO and favoring endothelial dysfunction.

On this regard, an in vitro study with cultures of human umbilical vein endothelial cells (HUVEC) demonstrated that the metabolite 3′-O-methyl (-)-epicatechin had the capacity to inhibit Nox, due to its structural similarities with apocynin (first known inhibitor of Nox) [26,53]. 

It has been determined that the presence of a mono methylated catechol ring is essential for the inhibitory effect of apocynin, 3′-O-methyl (-)-epicatechin and 4′-O-methyl (-)-epicatechin on Nox activity [54,55]. The latter was corroborated when incubation of 3′-O-methyl (-)-epicatechin in the presence of 3,5-dinitrocatechol (DNC), an inhibitor of catechol-O-methyltransferase (COMT), showed an increase in the production of O_2_^−^ and a decrease in the concentration of NO, due to its reaction with O_2_^−^ to form peroxynitrite (ONOO^−^) [53].

It has been suggested that epicatechin mono-O-methylated metabolites inhibit NADPH oxidase by affecting the assembly of the multi-protein complex (membrane-linked components and cytosolic proteins).

Oxidative stress also plays an important role in the pathogenesis of hypertension (HTA), which constitutes one of the main risk factors in the development and rapid progression of atherosclerosis. In a murine model of arterial hypertension (DOCA-salt HTA), the effect of two different doses of (-)-epicatechin on the activity of NADPH oxidase were studied. Results show that the chronic administration (5 weeks) of 10mg/kg of epicatechin significantly reduces the activity of this enzymatic complex, plasma markers of oxidative stress, and vascular production of O_2_^−^.

The proposed mechanism by which (−)-epicatechin inhibits Nox activity involves a decrease in the expression of its cytoplasmic subunit (p47_phox_), which acts as an adaptor protein that facilitates activation of gp91phox (membrane-bound subunit) [56].

Moreover, it has been shown that pretreatment with (-)-epicatechin suppresses the NADPH-oxidase-mediated generation of O_2_^−^ elicited by oxidized low-density lipoproteins (LDL) or angiotensin II in endothelial cells [54].

Previous in vitro studies studied the effect of other flavonoids on the vascular bioavailability of NO and the activity of endothelin-1 (ET-1). The latter is a powerful vasoconstrictor produced in the blood vessel walls that increases the production of O_2_^−^ through the endothelin receptor A (ETA)/NADPH oxidase pathway. Vascular dilatation is critically impaired by an upregulated ETA in association with a reducing nitric oxide bioavailability. In a study of vascular endothelial cultures, it was reported that green tea epigallocatechin-3-gallate (EGCG) stimulates the production of NO through the activation of the Fyn/PI3K/Akt/nitric oxide synthase and down-regulation of ET-1 gene and protein expression [57].

Phosphorylation and activation of Akt kinase (Ser^473^) and AMPK by EGCG are implicated in the phosphorylation and inhibition of FOXO1 (Thr^24^), which results in nuclear exclusion of this transcription factor and in its dissociation from hET-1 promoter [56,57].

Polyphenol compounds have demonstrated the capacity to inhibit endogenous antioxidant enzymes (superoxide dismutase (SOD), catalase (CAT), glutathione peroxidase (GPx)) [58]. The flavanols rich in cocoa revert N^G-^N-L-arginina methyl ester, which is an inhibitor of NO synthase, so that increases the production of NO (whose bioavailability is directly related to oxidative stress, because when it increases it is not converted to peroxynitrites) oxidants that reduce NO, having as a direct consequence vasoconstriction. The flavanols of cacao, due to their antioxidant properties, inhibit the production of peroxynitrites, increasing the bioavailability of NO and finally lowering blood pressure; these last effects are also produced by inhibiting the enzyme angiotensin converting enzyme, a key enzyme in the control of blood pressure, which inhibits the renin-angiotensin-aldosterone system [8]. 

##### Endothelial Nitric Oxide Synthase (eNOS) and Arginase

Inhibition of Nox is not the only mechanism proposed to explain cacao flavanols’ effect on increased bioavailability of NO. It has been reported that (-)-epicatechin can induce endothelial NO synthase (eNOS) activation through several mechanisms. eNOS is an homodimer binded by two calmodulins (CaMs) that present N-terminal oxigenase and C-terminal reductase domains. The first domain has binding sites for cofactors such as Zn, tetrahydrobioperin, heme group and L-arginin; while the second has biding sites for NADPH, flavin adenine dinucleotide (FAD) and Flavin mononucleotide (FMN) [18]. NO production is mediated by diverse NOS activities and its reaction with superoxide anion, on which its production and bioavailability depend. Exposure of vascular cells to high nanomolar levels or low micromolar concentrations of flavanols triggers a cellular response that indicates a potential existence of cellular surface aceceptors/effectors for epicatechin, which can mediate the subsequent activation of eNOS. This is associated with calcium homeostasis, both kinase II dependent and calmodulin-independent or increased through phosphorylation of serine residues [8]. 

L-arginine is a key substrate for eNOS endothelial production of NO. Limited availability of this amino acid changes the functional profile of eNos and instead of oxidizing L-arginine, the enzyme reduces molecular oxygen to O_2_^−^. The latter reduces NO bioavailability by rapidly reacting with it to form ONOO^−^ [59]. Vascular arginase competes with eNOS for their common substrate L-arginine. During inflammation and cellular oxidation, the increase in the concentration of arginase is linked to a decrease in the availability of L-arginine, a decreased synthesis of NO, an increase in ROS production, and endothelial dysfunction. 

Given the above, an increase in the expression and activity of arginase has been related to the progression of atherosclerosis, hypertension and cardiovascular diseases. An in vitro study showed that (-)-epicatechin and its structurally related metabolites lower arginase-2 expression and arginase activity (dose-dependent manner) in HUVEC. In accordance with these results, the consumption of a high-flavanol cocoa drink (985 mg/serving) showed a decreased arginase activity in human erythrocytes. Further in vivo evidence showed that high-flavanol containing cocoa-based diet (4% cocoa) lowers renal arginase-2 activity in rats [6,60].

##### Nuclear Factor κB (NF-κB) and Tumoral Necrosis Factor (TNF-α)

In view of the strong association between oxidative stress, chronic inflammation and metabolic syndrome, nuclear factor κB (NF-κB) has emerged as an important target to reduce chronic inflammatory response and development of non-communicable diseases. NF-κB is a ubiquitous heterodimeric protein (including c-Rel, RelB, RelA (p65), p50/p105 and p52/p100) that regulates the expression of a large family of genes that encode proteins involved in inflammation, innate immune response and regulation of cellular survival. It is also considered a useful marker of oxidative stress, since it is activated by ROS and pro-inflammatory cytokines (IL-1, IL-6, TNF-α). In most cells, NF-kB remains inactive in the cytoplasm when inhibitory IκB proteins bind to dimers of RelA, c-Rel, and p50. This interaction blocks the ability of NF-kB to act as a transcription factor. On the other hand, high levels of superoxide-derived ROS and proinflammatory cytokines (such as TNF-α and IL-1) lead to the activation of a specific IκB-kinase (IKK) complex that targets IκB for ubiquitination and proteasomal degradation (by phosphorylation on S32 and S36). The latter releases an active NF-κB that can translocate to the nucleus and bind to specific κB sites in select gene promoters to activate the transcription of genes involved in inflammation [48,61,62,63].

Procyanidin B2 and (-)-epicatechin can interfere in different levels of the NF-κB activation pathway, which plays a central role in the development of inflammation and regulator of the adhesion and expression molecules of cytokines. A decrease in cell oxidants through free radicals scavenging and inhibition of NADPH oxidase activity are two well known mechanisms that modulate the activation of NF-κB. Other mechanism that has been proposed is a previous inhibition of TNF-α. An in vitro study showed that pre-incubation of Jurkat T cells with increasing doses of procyanidin B1 and B2 reduces NF-κB activation mediated by TNF-α treatment. The expression of *IL-2* (NF-kB regulated gene) was also evaluated by measuring its release to the media. A decrease in its production, after treatment with procyanidin B1 and B2, suggested an inhibition of the formation of DNA/NF-κB complex. Evidence supported by a molecular model has shown that procyanidin B2 can interact with NF-kB proteins (RelA and p50) and prevent their binding to the DNA kB sites. The presence of certain OH groups in the B2 dimer allows the conformation of a folded structure that mimics the guanine pairs in the κB DNA sequence, which interact (forming hydrogen bonds) with the arginine residues of both p50 (Arg 54 and Arg 56) and RelA (Arg 33 and Arg 35) [34,48,62].

All of the above show that procyanidin B2 has the capacity to inhibit the union of NF-κB to DNA and thus decrease the expression of genes involved in oxidation and inflammation processes of the metabolic syndrome. Curcumin and resveratrol (found mainly in grapes and wines) have also been studied for their potential inhibitory effect on NF-κB activation and translocation to the nucleus in TNF-α-stimulated adipocytes. Evidence showed that when cells were co-incubated with TNF-α and either curcumin or resveratrol, degradation of IκB was inhibited, as well as NF-κB nuclear translocation. In accordance with these observations, it was demonstrated that both curcumin and resveratrol were able to down-regulate *TNF-α, IL-1β, IL-6*, and *COX-2* gene expression in a dose-dependent manner. Moreover, a significant reduction in secreted cytokine levels was also observed [64].

Considering the association between NF-κB activation and up-regulation of endothelial cell adhesion molecules expression, the effect of an acute consumption of a cocoa beverage (40 g of cocoa powder) on NF-κB activation was evaluated in human peripheral blood mononuclear cells (PBMC).

In this clinical trial, cocoa flavanols showed to reduce phosphorylation of p65, which is the transcriptional active subunit of NF-kB. A reduced activation of NF-kB resulted in lower concentrations of both inflammation markers: intercellular adhesion molecule (ICAM-1) and E-selectin. However, vascular cell adhesion protein-1 (VCAM-1) concentrations were not modified after the intervention. These results are in accordance with a previous study, where the consumption of dark chocolate decreased plasma levels of ICAM-1 but not of VCAM-1 [34].

##### Oxidized Low-Density Lipoproteins (LDLox)

An increase in the oxidation of LDL particles has been associated with low NO bioavailability and high production of ROS in the arterial wall.

The presence of oxidized LDL (oxLDL) constitutes a crucial factor in the development of atherosclerosis and vascular diseases. The latter is supported by evidence that shows that oxidized LDL promotes the formation of fatty streak lesions that give rise to the formation and progression of atherosclerotic lesions by increasing foam cell formation and by inducing inflammatory cytokines, chemokines and adhesion molecules production [65].

The increase in vascular oxLDL concentration has also been associated with an increase in proteosomal degradation of eNOS, thus inverting the eNOS/iNOS ratio. Epicatechin has been shown to protect endothelial cells against both oxLDL-mediated cytotoxicity and loss of eNOS protein [66].

Pretreatment of bovine aortic endothelial cells (BAEC) with (-)-epicatechin prevented oxLDL-elicited down-regulation of eNOS protein and partially the up-regulation of iNOS protein, thus shifting the eNOS/iNOS ratio toward preferencial expression of eNOS protein [67].

Furthermore, oxLDL-provoked oxidative stress in endothelial cells renders cellular proteins vulnerable to oxidative modification, which includes formation of protein-bound carbonyls and tyrosine-nitrated proteins with functional alterations. It has been observed that these modified proteins are mainly localized in the cytosol with highest concentrations at the border zone between cytosol and nucleus. In HUVEC, (-)-epicatechin (10 μM) showed a protective action on protein carbonyl and tyrosine-nitrated proteins formation elicited by oxLDL. Since elevated protein nitration is a consequence of increased iNOS-dependent nitrite formation, these results corroborate that (-)-epicatechin reduces the induction iNOS by oxLDL [67].

Table 2 summarizes the most relevant in vivo and in vitro studies demonstrating the antioxidant and anti-inflammatory effects and underlying mechanisms of cocoa and its main flavanols and procyanidins.

#### 4.2.2. Effects of Flavanols on Lipid Metabolism Disorders

According to the criteria for the diagnosis of the metabolic syndrome, the main lipid metabolism disorders include elevated plasma triglyceride (TG) levels and low concentrations of high-density lipoproteins (HDL-c).

Nevertheless, metabolic syndrome dyslipidemia also involves an increase in total cholesterol (TC), remnant chylomicrons, low-density lipoproteins (LDL-c) and very low-density lipoproteins (VLDL-c).

An overview of current clinical trials and in vitro studies evaluating cocoa and derived products consumption on metabolic syndrome lipid profile is summarized in Table 3.

Epidemiological evidence shows a direct relationship between the intake of polyphenol-rich vegetables and a lower predisposition to develop dyslipidemia and cardiovascular diseases. Observational studies and clinical assays in animals and humans have demonstrated the positive effects of cacao flavanols on oxidation, inflammation and endothelial function. It has also been highlighted that these compounds are able to improve the lipid profile that predisposes subjects to the development of atherosclerosis and cardiovascular diseases [70,71]. 

Meta-analysis of clinical assays have demonstrated that short term consumption of cacao-derived products (cocoa and dark chocolate) has benefits on the lipid profile of patients with some type of cardiovascular disease or with metabolic risk factors. Most of the studies are consistent when showing a decrease in the plasma levels of total (TC) and LDL-c. However, with regard to the increase in HDL levels, results are heterogeneous [70,71].

Other studies on animals and humans (healthy or with CV risk) have also reported a significant decrease in the plasma levels of TG, TC and LDL-c, as well as an increase in the HDL-c levels after chronic and acute consumption of cocoa powder or dark chocolate [27,72].

Additionally, supplementation with cocoa has shown a significant increase in the plasma concentrations of adiponectin, which has been related to a decrease in hepatic triglyceride content of mice and rats fed a high-fat diet [51,73].

An animal study evaluated the potential mechanisms for the hypocholesterolemic effect of a polyphenol extract from cocoa powder and a mixture of catechin (0.024%) and epicatechin (0.058%), after the ingestion of a high-cholesterol diet for 4 weeks. Results showed that the polyphenol extract group had significantly lower plasma cholesterol concentrations, and had significantly greater fecal cholesterol and total bile acids excretion than the mix (catechin/epicatechin) and the control group [74].

In vivo polyphenol extract effects were also assessed in vitro. It was shown that micellar solubility of cholesterol was significantly lower for procyanidin B2 (dimer), B5 (dimer), C1 (trimer) and A2 (tetramer) (main components of polyphenol extract) compared to catechin and epicatechin. These results indicate that oligomeric procyanidins from cocoa powder are the main active components responsible for the inhibition of cholesterol and bile acids intestinal absorption, through the decrease in micellar cholesterol solubility [75].

It has also been proposed that flavanols can act synergistically with tea polyphenols to increase their overall lipid-lowering effect through the inhibition of pancreatic lipase activity [83].

Chocolate intake has also been evaluated on the prevalence of the metabolic syndrome. The National Heart, Lung and Blood Institute (NHLBI) family heart study examined the association between self-reported chocolate consumption and the prevalence of MS in an adult US population. Results showed a lower prevalence of metabolic syndrome in groups with higher chocolate intake per week (>5 times/week) [71]. Table 3 shows that even when there is a big difference in the food matrix (powder or chocolate) or extracts, as well as in the concentrations tested; in addition if they are tested in humans, animals (obese or diabetic) or cell culture, consensus is found in the observed effects, that is, reduction of total cholesterol levels, triacylglycerols, both in serum and liver; in some cases reduction in glucose levels, lipoxidation in the kidney, and increase in insulin secretion were shown. In addition to observing these in humans, but without changes in the CRP. The inolucrated mechanisms, although already mentioned previously, the flavanols promote the phosphorylation of AMPK, in adipose tissue, by which the translocation of GLT4 is improved, in addition to increases in the gene and protein expression of UCP1 and UCP2 [76,77,78,79,80,81,82].

The hypocholesterolemic effects of apple procyanidins have also been studied. In rats fed a purified diet containing 0.5% cholesterol, the supplementation with apple procyanidins (AP: 0.2%, 0.5% and 1%) showed a decrease in liver and serum TG and TC levels in a dose-dependent manner compared with the control group. Additionally, the levels of HDL-c were significantly higher in the groups supplemented with AP 0.5% and 1% compared to the control group

In the liver, AP at levels of 0.5% and 1% significantly lowered cholesterol levels. This effect was associated with the modulation of hepatic cholesterol 7alpha-hydroxylase (CYP7) activity and an increased fecal excretion of acidic steroids [84].

A similar study evaluated the anti-obesity effects of apple and tea procyanidins. Supplementation of a high-fat diet (HF) with 1% of tea or apple procyanidins showed lower total white adipose tissue levels compared to the control group (HF alone). This result was in accordance with a decrease in serum leptin levels by dietary procyanidins (tea and apple). However, no changes were detected in serum adiponectin and insulin levels.

On the other hand, serum and hepatic TG levels were lowered by dietary procyanidins compared to the control group. In contrast, tea and apple procyanidins reduced serum TC levels, but showed no effect on liver TC levels.

It was suggested that these effects were mediated by a combination of an agonist-like action of PPARα and a moderate antagonist-like action of PPAR-γ, which in turn promoted a decrease in hepatic fatty acid synthase (FAS) activity and an increase in the peroxisomal β-oxidase activity [84].

A reduction on intrahepatic TG and cholesterol levels have been attributed in part to the modulation in the expression of genes related to their metabolism. In vitro, the acute administration of grape seed procyanidins decreased the expression of 3-hydroxy-3 methyl-glutaryl-CoA reductase (HMG-CoA-reductase), the limiting enzyme in the biosynthesis of cholesterol.

In the liver, lipid metabolism disorders promote an increase in fat infiltration and lipotoxicity that result in the development of non-alcoholic steatohepatitis (NASH), one of the most severe comorbidities of the metabolic syndrome [65,77].

An experimental study evaluated the preventive effects that cocoa supplementation may have on the development of NASH induced by a high-fat and choline deficient diet. Results showed that cocoa supplementation reduces the degree of hepatic steatosis, hepatic fibrosis and portal inflammation.

It was suggested that these effects could be mediated in part by up-regulation of gene and protein expression of the liver fatty-acid binding protein (LFABP) in rats with NASH. LFABP prevents non-alcoholic fatty liver disease (NAFLD) development by shuttling long-chain fatty acids towards the mitochondria for β-oxidation and possibly by acting as an antioxidant when intracellular antioxidants (CAT, SOD, GPx) are insufficient [77].

Effects of catechin, epicatechin and procyanidinB2, as well as extract of cocoa rich in flavanols on lipid metabolism disorders have also been studied. In an animal study, the effects of supplementing and hyperlipidic diet (21.2% of fat), 10 mg/Kg body weight of the pure compounds and 100 mg of the extract during eight weeks were evaluated. Results showed that flavanols supplementation reduced the accumulation of intrahepatic TG and fatty acids, thus inhibiting the development of steatosis. The proposed underlying mechanisms were the down-regulation of hepatic lipogenic genes expression (fatty acid synthase, PPAR-ɣ, and Cd36), as well as an up-regulation of lipolytic genes expression (PPARα, PGC1α, SIRT1) [85]. These results agree with those observed in vivo with vaticanol C (a resveratrol tetramer), which showed to be a more potent activator for PPARα than resveratrol. Vaticanol C also showed to induce PPARα-dependent genes, such as hepatic fatty acid binding protein-1 (FABP1) and acyl-CoA oxidase-1 (Acox1) [86].

Naringin is an active flavanone glycoside present in cocoa although at low concentrations that showed a significant lipid-lowering effect in a mouse model fed with a high-fat diet for 20 weeks. Supplementation with this compound (0.2 g/kg) improved dyslipidemia and hepatic steatosis by reducing serum and liver cholesterol levels. However, it did not reduce liver TG levels. Additionally, in white adipose tissue, naringin treatment reversed hypertrophic adipocytes. Among the mechanisms underlying naringin hypolipidemic effects were an up-regulation of four genes involved in fatty acid oxidation (*PPARα, CPT-1a, ACOX, UCP2*), and a down-regulation of three genes encoding enzymes involved in fatty acid synthesis (*SERBP-1c, FAS, ACC*). Stearoyl-CoA desaturase-1 (SCD-1), a very important lipogenic enzyme responsible for triglyceride metabolism, showed no alteration after naringin treatment. The latter explains why naringin was not able to reduce liver TG levels [87].

#### 4.2.3. Effects on Hyperglycemia and Insulin Resistance

Besides the antioxidant, anti-inflammatory and hypolipidemic effects demonstrated by cacao flavanols, publications have emphasized their potential benefits in reducing hyperglycemia, insulin resistance and diabetes (Table 3). The latter are closely related to dyslipidemia, accumulation of abdominal fat and to the pathogenesis of the metabolic syndrome.

The effect of flavanol-rich dark chocolate intake (110.9 mg epicatechin/100 g) on endothelial function, insulin sensitivity, β-cell function and blood pressure was evaluated in hypertensive patients with impaired glucose tolerance. After 15 days of dark chocolate intake, an increase in vasodilation was observed (by increasing NO availability), insulin sensitivity and β-cell function, as well as a decrease in BP and hyperglycemia [81,88].

In vivo studies have shown that the administration of cacao liquor rich in procyanidins (CLPr) to diabetic and obese mice decreased hyperglycemia in a dose-dependent manner. The mechanisms proposed for this effect involved an increase in the translocation of GLUT4 towards the cell membrane, an increase in the phosphorylation of AMPK and the up-regulation of *UCP-2* gene expression in the skeletal muscle [78,82]. These results agree with those obtained by Ruzaidi et al, in which the administration of a cocoa extract in a DM 2 animal model, resulted in hypoglycemic (reduction of plasma glucose levels and insulin mimicking activity) and hypolipidemic effects [79,80].

In a further study, the effect of supplementing a standard diet with different concentrations of CLPr was evaluated in obese female rats with DM2. Results showed that groups fed a standard diet containing 0.5% and 1% CLPr had lower glycemic levels from the first week of the treatment as compared to the control group. It is noteworthy that this effect was dose-dependent [78]. A similar study evaluated if supplementation with CLPr was able to attenuate the development of obesity, insulin resistance and hyperglycemia induced by a high-fat diet. At the end of the experiment, it was observed that different doses of CLPr decreased the levels of fasting plasma glucose as compared to the group fed with a high-fat diet without supplementation. Oral glucose tolerance was examined in order to evaluate the effects of CLPr on postprandial hyperglycemia. Results indicated that supplementation with 2% CLPr suppressed postprandial hyperglycemia and hyperinsulinemia [82].

In this study, it was also confirmed that the molecular mechanisms involved in CLPr hypoglycemic effects were the activation of AMPK and an increase in GLUT4 translocation. Finally, the effect of the CLPr supplementation on the regulation of thermogenesis and energy metabolism was also studied [82].

Results showed that both concentrations of CLPr (0.5 AND 2%) increased energy expenditure through up-regulation of gene and protein expression of UCP1 (expressed in brown adipose tissue) and UCP2 (expressed in white adipose tissue and liver) [82]. These results agree with those obtained [87], in which the effect of supplementing a high-fat diet with naringin (0.2 g/kg of body weight) was evaluated in a murine model of diet-induced obesity.

Results showed a significant decrease in fasting plasma glucose and insulin levels, which indicated a relevant improvement in insulin sensitivity among different tissues.

In animal models of obesity, low plasma adiponectin levels and high plasma TNF-α levels were observed. Supplementation with naringin decreased plasma TNF-α levels and increased those of adiponectin [87]. Besides, it was observed that through the activation of the AMPK pathway, naringin can down-regulate glycogenic enzymes gene expression (PEPCK and G6Pase), and thus increase glucose uptake by an insulin-independent pathway [87]. Treatment of rat hepatic cell cultures with epigallocatechin-3-gallate has been shown to induce insulin receptor and insulin receptor substrate (IRS-1) tyrosine kinase activity and to down-regulate phosphoenlopyruvate carboxykinase gene expression (a key enzyme in gluconeogenesis) [89].

## 5. Conclusions

In summary, there is evidence from in vitro assays, animal experiments and human intervention trials, which demonstrate that cacao flavanols have the potential to modulate several risk factors associated with the development of the metabolic syndrome.

Considering that flavanols’ bioactivity in vivo depends on their absorption and metabolism, there has been a great advance in the analysis of cocoa flavanols metabolites in plasma and urine (mainly from (-)-epicatechin) after the administration of pure compounds or the consumption of cocoa or dark chocolate. 

However, further studies on cellular uptake of O-methylated, O-glucuronidated, and O-sulphated forms are required in order to provide information on which metabolites are the most able to enter cells. The latter will be useful to predict their localizations in different body tissues.

On the other hand, since cocoa is a complex mixture of monomeric and oligomeric flavanols, future experiments should be focused on the possible interactions between flavanols in the gastrointestinal milieu and their synergic effects on their bioavailability. Moreover, it would be important to study the possible effects that the presence of different macronutrients (proteins, lipids and sugars) in cocoa-derived products could have on flavanols absorption. 

Additionally, we have shown some of the potential molecular targets of cacao flavanols and the proposed mechanisms responsible for their alleviating effects on metabolic syndrome development. 

Further studies on cocoa flavanols’ effects should include their major metabolites (at a physiologically achievable dose), in order to reveal if they are linked to the biological effects and underlying mechanisms of cocoa products.

Together, all this information will serve as the basis for a careful review to provide meaningful dietary recommendations for the consumption of cocoa flavanols.

## Figures and Tables

**Table 1:** Flavanols bioavailability.

Type of Study	Product/Compound		Dose	Plasma Metabolites	Plasma Cmax (μmol/L)	Plasma Tmax (H)	Area Under Curve AUC	Urinary Excretion	T ½	Reference
In vivo Sprague–Dawley male rats (*n* = 30)	(-)-epicatechin		1, 5 and 10 mg/kg	Total 3′-O-methylated forms (conjugated + no conjugated)	1 ± 0.02; 3.05 ± 0.15; 4.5 ± 0.22	1	-	Total (-)-epicatechin nonmethylated and 3′-O-methylated metabolites (nM/18 h): 397 ± 35 nM; 1870 ± 101 nM; 3003 ± 212nM	-	[27]
				Total nonmethylated forms	0.97 ± 0.14; 3.21 ± 0.29; 4.41 ± 0.50					
	cocoa poder		150, 750 and 1500 mg/kg	Total 3′-O-methylated forms (conjugated + no conjugated)	0.12 ± 0.04; 1.05 ± 0.05; 2.49 ± 0.16	1	-	Total (-)-epicatechin metabolites (non-methylated and 3′-O-methylated): 415±18 nM; 1523±120 nM; 3074±218 nM/18 h	-	
				Total nonmethylated forms	0.35 ± 0.04; 2.12 ± 0.05; 5.08 ± 0.43					
In vivo Sprague–Dawley male rats (*n* = 20)	(-)-epicatechin		172 μmol/kg	Total 3′-O-methylated forms	-	-	78.3 ± 4.9 μmol.h/L	9.45 ± 0.56 μmol/24 h	-	[28]
				Total non-methylated forms			88.3 ± 12.4 Μm ol.h/L	16.6 ± 2.3 μmol/24 h		
	(+)-catechin		172 μmol/kg	Total 3′-O-methylated forms	-	-	23 ± 1.1 μmol.h/L	3.60 ± 0.07 μmol/24 h	-	
				Total non- methylated forms			66.4 ± 2.8 μmol.h/L	8.85 ± 0.76 μmol/24 h		
	Mix		345 μmol/kg	Total epicatechin 3′-O-methylated forms	-	-	76.5 ± 6.8 μmol.h/L	4.51 ± 0.45 μmol/24 h	-	
				
				Total epicatechin nonmethylated forms			78.7 ± 4 μmol.h/L	9.43 ± 0.58 μmol/24 h		
				Total catechin 3′-O-methylated forms			18.9 ± 0.4 μmol.h/L	2.53 ± 0.34 μmol/24 h		
				Total catechin nonmethylated forms			56.5 ± 3.5 μmol.h/L	7.21 ± 0.51 μmol/24 h		
In vivo Wistar albino male rats	[^14^ C] procyanidin B2	21 mg/kg IV		-	-	AUC_(0__−__24)_: 149 ± 21μg.h/min	75.6 ± 5.4 % of total dose/24 h	6.67 ± 0.95		[23]
		21 mg/kg IG		2.60 ± 0.93 μg/Ml	6.11 ± 0.43	AUC_(0−24)_: 17 ± 2.7μg.h/min	62.9 ± 5.48 % of dose	7.3 ± 2.07		
		10.5 mg/kg IG		1.38 ± 0.28 μg/mL	5.56 ± 0.98	AUC_(0__−__24)_: 5.18 ± 1.35μg.h/min	62.2 ± 7.6 % of dose	4.57 ± 1.46		
			(−)-epicatechin-3′-sulfate; (−)-epicatechin-5-sulfate; (−)-epicatechin-7-sulfate	331 ± 26 nM; 37 ± 3 nM; 12 ± 1 nM assessed using authentic standards	2					
			Unmetabolized (−)-epicatechin	4 ± 1 nM	1					
Healthy volunteers (*n* = 5; 23.47 ± 3.3 years)	100g of Nestle’ Noir 70% chocolate	Content: 79mg (-)-epicatechin 26mg (+)-catechin 49mg procyanidin B2	(-)-epicatechin-3′-β-D-glucuronide; (-)-epicatechin-4′-β-D-glucuronide; (-)-epicatechin-7-β-D-glucuronide	290 ± 49 nM; 44 ± 11 nM; 22 ± 6 nM	3.2 ± 0.2; 3.4 ± 0.3; 12.8 ± 4.8	1276 ± 182 nM/h; 164 ± 38 nM/h; 360 ± 50 nM/h	13.3 ± 3.85 μmol/24 h; 1.03 ± 0.06 μmol/24 h; 7.27 ± 1.35 μmol/24 h	3.8 ± 1.0; 1.8 ± 0.3; 5.6 ± 1.1		[30]
			(-)-epicatechin 3′-sulfate; (-)-epicatechin 4′-sulfate	233 ± 60 nM; 11 ± 3nM	3.2 ± 0.2; 3.5 ± 0.3	954 ± 207 nM/h; 66 ± 8 nM/h	8.53 ± 2.71 μmol/24 h; 0.56 ± 0.13μmol/24 h; (-)-epicatechin 5-sulfate: 1.15 ± 0.20μmol/24 h	2.3 ± 0.8; 4.1 ± 0.9;		
			3′-O-methyl-(-)-epicatechin 4′-sulfate; 3′-O-methyl-(-)-epicatechin 5-sulfate; 3′-O-methyl-(-)-epicatechin 7-sulfate; 4′-O-methyl- (-)-epicatechin 5-sulfate 4′-O-methyl-(-)-epicatechin 7-sulfate	49 ± 14 nM; 153 ± 43 nM; 40 ± 10 nM; 18 ± 6 nM; 13 ± 4 nM	3.6 ± 0.3; 3.8 ± 0.2; 3.8 ± 0.2; 3.8 ± 0.3; 3.8 ± 0.2	269 ± 74 nM/h; 679 ± 160 nM/h; 222 ± 59 nM/h; 94 ± 19 nM/h; 70 ± 22 nM/h	1.67 ± 0.62 μmol/24 h; 14.1 ± 3.88 μmol/24 h; 2.33 ± 0.68 μmol/24 h; 1.37 ± 0.34 μmol/24 h; 0.73 ± 0.23 μmol/24 h	2.5 ± 0.6, 2.1 ± 0.6; 2.1 ± 0.6; 2.3 ± 0.5; 2.0 ± 0.8		

**Table 2:** Flavanols: antioxidant and anti-inflammatory effects.

Type of Study	Product/Compound	Dose/Duration	Intervention	Target	Outcome (S)	Reference
2-year-old male Wistar rats (*n* = 48)	(-)-epicatechin	2 and 10 mg/kg bw intragastric administration, during 5 weeks	DOCA-salt induced hypertension vs. DOCA-salt EPI2 and DOCA-salt EPI10	Vascular Nox activity Protein expression of Nox p47^phox^ and p22^phox^ subunits	DOCA-salt–EPI10 ↓ Nox activity in aortic rings by suppression of protein over-expression of p47^phox^ and p22^phox^ subunits and ↓ in ET-1 plasma levels Both DOCA-salt–EPI2 and EPI10 restored impaired endothelial function due to an ↑ in eNOS phosphorylation and a ↓ in O_2_^−^ vascular content	[56]
Double blind study with crossover-design in healthy volunteers (*n* = 10)	High-flavanol cocoa beverage (98 mg total flavanols: 183 mg epicatechin and 215 mg dimers) Low-flavanol cocoa beverage (80.4 mg total flavanols: 19.8 mg epicatechin and 23.1 mg dimers)	54 g/200 mL of high or low-flavanol cocoa beverage	High-flavol cocoa (HFC) vs. low-flavanol cocoa (LFC)	Erythrocyte arginase activity	Ingestion of a high-flavanol cocoa beverage resulted in the highest decrease in erythrocyte arginase activity after 24 h (HFC: 3.0± 0.4; *p* < 0.05 vs. LFC: 3.5 ± 0.5 μmol urea mg protein-1 h-1)	[60]
Jurkat T cells culture HCAEC (human coronary artery endothelial cells) culture Obesity mice 2 months old C57BL	Procyanidin A1, procyanidin A2, procyanidin B1 and procyanidin B2 (-)-epicatechin	Cells (1×10^6^ cells/ml) were pre-incubated with 2.5–50 μM A1, A2, B1 or B2 for 24 h Incubation of 0.1 nM-100 μmol/L during 10 minutes 1 mg epicatechin/kG Body weight 15 days	Effect of preincubation of Jurkat T cells (further incubation with or without the addition of either TNF-α or PMA) Identification of epicatechin intracellular signaling pathways on eNOS-NO production Inflammatory status: TNF αand IL-6	NF-κB-DNA binding eNOS activation	Pre-incubation (24 h) with B1 or B2 procyanidins (50 μM) ↓ NF-κB-Luc activity (34–52%) and ↓ by 80 and 85% IL-2 release in Jurkat cells subsequently treated with TNF-α or PMA A concentration-dependent (5-50μM) inhibition of NF-κB-DNA binding was observed in cells pre-incubated with B1 or B2 procyanidins At 100 nM, B1 and B2 caused a 29–38% and 38–47% inhibition of either p50 or RelA binding to its DNA consensus sequence Epicatechin (1 μM/L) induced eNOS activation via Ser1177 and Ser633 phosphorylation and Thr495 de-phosphorylation Epicatechin (1 μM/L) activated eNOS via Akt phosphorylation (induction of Ser1177 phosphorylation) Epicatechin stimulated dissociation of eNOS from Cav-1 and therefore stimulated its activation TNF α level decreased by 50 % while IL-6 decreased by 30%	[62] [68] [69]
RAEC, BAEC and human umbilical endothelial cells (HUVEC) cultures	(-)-epicatechin	20 μM incubation for 24 h	Protective effects of (-)-epicatechin against oxLDL protein damage	NADPH oxidase (NOX) activity and oxLDL protein damage	Pretreatment of BAEC and RAEC with epicate-chin prevented oxLDL-elicited downregulation of eNOS protein and par-tially the upregulation of iNOS protein In BAEC and HUVEC incubated with oxLDL, (-)-epicatechin showed a potent O_2_^−^ scavenging activity and a strong inhibition of its production (10 μM) Pretreatment of HUVEC with (-)-epicatechin su-ppressed the formation of all 3 types of modified proteins (protein carbo-nyls and tyrosine-nitrated proteins) in a dose-dependent manner (com-plete inhibition at 10 μM)	[67]
HUVEC culture	(-)-epicatechin, its metabolites (3′-O-methyl epicatechin, 4′-O- methyl epicatechin) and pB2	0.1-100 μM incubation for 24 h	Effect of pB2, epicatechin and its metabolites on NADPH oxidase activity	NADPH oxidase (NOX) activity and O_2_^−^ generation	All 4 compouds (10 μM) inhibited O_2_^−^ re-lease in Angiotensin-II estimulated HUVEC, after 24 h preincubation Methylated epicatechin metabolites proved to be Nox inhibitors (100 μM) without O_2_^−^ scavenging activity Epicatechin showed O_2_^−^ scavenging activity (100 μM) dependent on the duration of preincuba-tion, but did not affect NOX oxidation pB2 showed both inhibitory Nox and O_2_^−^ scavenging activities	[54]
Sprague–Dawley male rats (*n* = 10)	Cocoa powder (11 mg epicatechin/g and 43 mg procyanidins/g)	Purified egg white protein-based diet containing 40 g cocoa/kg diet, during 28 days	Diet 0% cocoa vs. Diet 4% cocoa	Renal arginase activity	4% cocoa supplementation ↓ renal arginase activity, compared with control group (0.13 ± 0.02 vs. 0.18 ± 0.02 U/mg protein)	[60]
HUVEC culture	(-)-epicatechin flavanol metabolite mixture (2.6 μM total flavanols: 0.1 μM epicatechin and 2.15 μM epi-catechin metaboli-tes found in human plasma 2 h after high-flavanol cocoa beverage consumption)	mix: 0.4, 2.6 and 7.8 μM epicatechin: 1, 3 and 10 μM 48 hour incubation	Comparison between different concentrations of flavanol mix and epicatechin	Arginase-2 (Arg-2) mRNA expression and activity	Flavanol mix and epicatechin signifi-cantly ↓ Arg-2 mRNA expression in HUVEC, at 24 h in a dose-dependent manner Cells incubated with flavanol mix and epica-techin exhibited ↓ Arg-2 activity, at 48 h in a dose-dependent manner	[60]
Randomized, crossover clinical trial in healthy volunteers (*n* = 18)	Cocoa powder	40 g cocoa powder (28.2mg epicatechin and 25.5 mg pB2/40 g) with 250 mL whole milk or water, during 3 weeks	Cocoa powder with milk (CM) vs. cocoa powder with water (CW)	NF-κB activation and protein expression of adhesion molecules (sICAM-1, sVCAM-1 and sE-selectin) in PBMC (periphe-ral blood mono-nuclear cells)	CW significantly ↓NF-κB activation (determined by protein expression) after 6 h of ingestion, compared with CM Both CM and CW ↓ serum [sICAM-1] after intervention but only CW ↓ [sE-selectin]	[34]
Human hepatoma HepG2,	(-)-Epicatechin (EC) and cocoa phenolic extract (CPE)	10 µM EC or 1 µg//mL CPE were added to the cells for 24 h;	Comparison between epicatechin and polyphenol extract	Nrf2; GPx, GX and CAT	Antioxidant exnzymes were regulating and Nfr2 has been stimulated.	[46]

bw: body weight; DOCA-salt: deoxycorticosterone acetate and sodium chloride; EPI: (-)-epicatechin; Nox: NADPH oxidase; ET-1: endothelin-1; eNOS: endothelial nitric oxide synthase; O_2_^−^: superoxide; NO: nitric oxide; oxLDL: oxidized LDL; RAEC: rat aortic endothelial cells; BAEC: bovine aortic endothelial cells; iNOS: inducible nitric oxide synthase; pB2: procyanidin B2; NF-κB: nuclear factor κB; PMA: phorbol myristate acetate; TNF-α: tumor necrosis factor-alpha; Cav-1: caveolin-1. ↑ increased; ↓decreased.

**Table 3:** Flavanols: hypolipidemic and hypoglycemic effects.

Type OF Study	Product/Compound	Dose/Duration	Intervention	Target	Outcome (s)	Reference
Comparative, double-blind study in normo and mild hyper cholesterolemic japanese subjects (*n* = 160)	Low PFT cocoa powder (64.5 mg epicatechin and 36.3 mg pB2/g) middle PFT cocoa powder (96.7 mg epicatechin and 54.4 mg pB2/g) high PFT cocoa powder (129 mg epicatechin and 72.5 mg pB2/g)	Consumption of 13 g low PFT cocoa; or 19.5 g middle PFT cocoa; or 26 g high PFT cocoa, during 4 weeks	Intake of low PFT cocoa powder vs. middle PFT cocoa vs. high PFT cocoa in normo and mild hyper cholesterolemic subjects	Serum LDL, HDL and oxLDL	Consumption of 3 cocoa doses in subjects with LDL ≥3.23 mmol/L, resulted in significantly ↓ serum [LDL], after 4 wk Consumption of 3 cocoa doses (normo and mild hypercholesterolemic subjects) resulted in ↑ serum [HDL], compared with baseline after 4 wk Plasma oxLDL levels were significantly ↓ after 4 wk consumption of 3 cocoa doses (normo and mild hyper cholesterole-mic subjects)	[72]
Randomized, placebo-controlled, double blind, crossover study in DM 2 subjects (*n* = 12)	High (16.6 mg epicatechin) and low (<2 mg epicatechin) polyphenol content chocolate	45 g high or low polyphenol chocolate, during 8 week	High polyphenol chococolate (HPC) intake vs. low polyphenol chococolate (LPC) intake	Serum c-HDL, c-LDL, TG, HbA1c, fasting glucose and insulin and C- reactive protein	Consumption of HPC and LPC improved lipid profile through ↑ HDL/↓ LDL No changes were observed in fasting glucose or HbA1c levels in none of the 2 treat-ment groups Insulin levels showed an ↑ after LPC intake No changes were observed in C-reactive protein levels in none of the 2 treatment groups	[75]
Cross-sectional study in 4098 patients from NHLBI	Chocolate	-	Association between self-reported chococlate consumption and prevalence of metabolic syndrome (MS) in adult population	ATP-III criteria for clinical diagnosis of metabolic syndrome	Higher intake of chocolate was associated with ↓ prevalence of coronary heart disease and ↓ glycemia From the lowest to the highest levels of choco-late consumption, the prevalence of MS odd ratios were: + women: 1.0 (0/wk); 1.26 (<1/wk); 1.15 (1–4/wk) and 0.9 (+5/wk) + men: 1.0 (0/wk); 1.13 (<1/wk); 1.02 (1-4/wk) and 1.21 (+5/wk) the highest odd ratios of obesity prevalence were observed with higher chocolate consumption	[71]
9-week-old male Sprague–Dawley rats (*n* = 40) 12-week-old female Sprague–Dawley rats (*n* = 56)	Cacao procya-nidins (CP) extracted from cacao liquor (CLPr: 79.3% total polyphenols; 5.9% epicatechin; and 4% PB2) Cocoa powder	High-cholesterol diet (HCD: 1% cholesterol and 15% fat) supplemented with 0.5 or 1.0% of CLPr C1: methionine-choline deficient diet (MCD) + 28 d of 12.5% cocoa supplementation C2: MCD diet + 56 d of cocoa supplementation C3: 80 d of MCD + cocoa supp. C4: 108 d of MCD + cocoa supplementation	HCD with 0.5% CP vs. HCD with 1.0% CP C1 and C2 were selected to test NASH treatmet effects of cocoa supplementation C3 and C4 were used to test if cocoa supple- mentation could prevent NASH development	Plasma and liver cholesterol liver and feces TG mRNA and protein expression of LFABP serum TG, glucose and superoxide levels	Both CP groups (0.5 and 1%) inhibited drastic elevation of plasma TC levels Liver cholesterol and TG levels were significantly ↓ in HCD groups supplemented with both CP doses (more marked effects in 1% CP) All cocoa supplemented groups showed ↓ serum TG and glucose levels C3 group had the ↓ superoxide levels, compared to C1, janeC2 and C4 groups C1 had the ↑ mRNA and protein expression levels of LFABP	[76] [77]
3-week-old female diabetic obese mice (*n* = 44) Streptozotocin-diabetic male Wistar rats (*n* = 80: 200–300 g)	Cacao liquor procyanidins (72.32% total polyphenols; 5.89% epi-catechin and 3.93% PB2) Cocoa beans extract (CE)	Supplementation with 0.5 or 1 % CLPr, during 3 weeks Supplementation with 1, 2 or 3% CE (1 g CE/100 g diet)	Dietary supplementation with 0% CLPr vs. supplementation with 0.5 or 1.0% CLPr Streptozotocin-diabetic rats + normal diet vs. diabetic induced rats + 1, 2 or 3% CE	Plasma glucose (hyperglycemia) and renal function Serum CT, HDL, LDL, TG and glucose	Levels of blood glucose were significantly ↓ in mice fed 1% CLPr At the end of the study, group supplemented with 1% CLPr had ↓ levels of BUN and creatinine and suppressed membrane lipoxidation in kidney (↓ 4-hidroxy-2-nonenal antibody levels) All 3 diabetic groups treated with CE showed significant ↓ in body weight gain and serum TG levels Diabetic groups treated with 1 and 3% CE exhibited significant ↓ in plasma glucose levels Diabetic group treated with 1% CE had the ↓ serum levels of CT and LDL	[78] [79]
Male Sprague–Dawley rats (n= 90)	Polyphenol-rich cocoa extract (CE)	Intragastric administration of 1, 2 or 3% CE (1 mL/100 g bw) during 4 weeks	Assessment of CE effectiveness in reducing hyperglyce-mia in diabetic-induced rats	Plasma glucose levels and body weight gain	A significant body weight reduction was observed (p < 0.05) in diabetic-induced rats treated with 1 and 2% CE Diabetic-induced rats treated with 3% CE showed the most significant ↓ in glucose levels	[80]
Glucose-responsive pancreatic cell lines (BRIN-BD11) Randomized, crossover trial in patients with hypertension (HTA-I) and impaired glucose tolerance (IGT) (*n* = 19)	Polyphenol-rich cocoa extract (CE) Flavanol-rich dark chocolate bar (FRDC: 110.9 mg epicatechin/bar)	Incubation with CE at 2, 1, 0.5, 0.1 and 0.05 mg/mL 100 g of dark chocolate bar, during 15 days	Evaluation of different concentrations of CE on insulin secretion Flavanol rich dark chocolate bar vs. flavanol free chocolate bar	Insulin-release from rat pancreatic β-cells Fasting glucose and insulin sensitivity Serum C-reactive protein (CRP) Serum lipid profile (HDL, CT, LDL and TG)	Pancreatic cell lines treated with 0.1 mg/mL of CE showed the ↑ insulin secretion FRDC intake enhanced insulin sensitivity and β-cell function in HTA-I patients with IGT (measured by ↑ QUICKI; ↓ HOMA-IR; ↑ ISI0 and ICI20) FRDC ingestion significantly ↑ FMD FRDC intake ↓ serum CT and LDL, but did not affect TG and HDL neither FRDC nor FFWC ingestion affected serum CRP	[81]
Male C57BL/6 4-week-old mice (*n* = 36)	cacao liquor procyanidin extract (CLPr: 6.12% epicatechin and 3.60% PB2)	Supplementation with 0.5 or 2% CLPr, during 13 weeks	High fat diet (HFD) vs. HFD + 0.5% (HF-0.5) or 2% (HF-2) CLPr	Glucose parameters mRNA and protein expression of UCP-1, UCP-2, GLUT-4 and AMPKα	At week 7, fasting glucose levels in HF-2 group were significantly lower At week 11, OGTT^e^ showed ↓ glucose levels in HF-2 group (0 and 15 minutes after glucose load) At the end of the study, HF-0.5 and HF-2 completely suppressed HF diet-induced hyper-glycemia, hyper-insulinemia (↓ HOMA-IR) and hypercholestero-lemia, compared to control group CLPr supplementation promoted AMPKα phosphorilation (BAT, WAT, liver and skeletal muscle), which enhanced GLUT-4 translocation to plasma membrane in BAT and skeletal muscle in a dose-dependent manner CLPr ↑ UCP-1 and UCP-2 gene and protein expression in BAT and WAT, respectively	[82]

PFT: total polyphenols; OGTT: oral glucose tolerance test; oxLDL: oxidized LDL; HbA1c: glycated hemoglobin; NHLBI: National Heart, Lung, and Blood Institute; NASH: non-alcoholic steatohepatitis; LFAP: liver fatty acid binding protein; bw: body weight.↑ increased; ↓decreased.

## References

[1] Rangel-Fajardo M.A., Zavaleta-Mancera H.A., Córdova-Téllez L., López-Andrade A.P., Delgado-Alvarado A., Vidales-Fernández I., Villegas-Monter Á. (2012). Anatomía e histoquímica de la semilla del cacao (*Theobroma cacao* L.) criollo mexicano. Anatomy and histochemistry of the mexican cacao (*Theobroma cacao* L.) seed. Rev. Fitotecnia Mex.

[2] Colombo M.L., Pinorini-Godly M.T., Conti A., Conti A., Paoletti R., Poli A., Visioli F. (2012). Botany and Pharmacognosy of the Cacao Tree. Chocolate and Health.

[3] Martín M.A., Ramos S. (2016). Cocoa polyphenols in oxidative stress: Potential health implications. J. Funct. Foods.

[4] Redovniković I.R., Delonga K., Mazor S., Dragović-Uzelac V., Carić M., Vorkapić-Furač J. (2009). Polyphenolic content and composition and antioxidative activity of different Cocoa liquors. Czech J. Food Sci..

[5] Bauer S.R., Ding E.L., Smit L.A. (2011). Cocoa Consumption, Cocoa Flavonoids, and Effects on Cardiovascular Risk Factors: An Evidence-Based Review. Curr. Cardiovasc. Risk. Rep..

[6] Sudano I., Flammer A.J., Roas S., Enseleit F., Ruschitzka F., Corti R. (2012). Cocoa, blood pressure, and vascular function. Curr. Hypertens. Rep..

[7] Loffredo L., Violi F. (2013). Polyphenolic antioxidants and health. Choc. Heal..

[8] Wang Y., Feltham B.A., Suh M., Jones P.J.H. (2019). Cocoa flavanols and blood pressure reduction: Is there enough evidence to support a health claim in the United States?. Trends Food Sci. Technol..

[9] Corella D., Ordovas J.M. (2007). Metabolic syndrome pathophysiology: The role of adipose tissue. Nutr. Metab. Cardiovasc. Dis..

[10] Ritchie S.A., Connell J.M.C. (2007). The link between abdominal obesity, metabolic syndrome and cardiovascular disease. Nutr. Metab Cardiovasc. Dis..

[11] Srikanthan K., Feyh A., Visweshwar H., Shapiro J.I., Sodhi K. (2016). Systematic review of metabolic syndrome biomarkers: A panel for early detection, management, and risk stratification in the West Virginian population. Int. J. Med. Sci..

[12] Alberti K.G.M., Zimmet P., Shaw J. (2006). Metabolic syndrome—A new world-wide definition. A Consensus Statement from the International Diabetes Federation. Diabet. Med..

[13] Trivedi R., Patel P.N., Jani H.J., Trivedi B.A. (2011). Nutrigenomics: From molecular nutrition to prevention of disease. Biotechnol. Indian J..

[14] Espín J.C., García-Conesa M.T., Tomás-Barberán F.A. (2007). Nutraceuticals: Facts and fiction. Phytochemistry.

[15] Strat K.M., Rowley T.J., Smithson A.T., Tessem J.S., Hulver M.W., Liu D., Davy B.M., Davy K.P., Neilsonc A.P. (2016). ScienceDirect Mechanisms by which cocoa flavanols improve metabolic syndrome and related disorders ☆. J. Nutr. Biochem..

[16] Rusconi M., Conti A. (2010). Theobroma cacao L., the Food of the Gods: A scientific approach beyond myths and claims. Pharmacol. Res..

[17] Braicu C., Irimie A., Berindan, Pilecki V., Balacescu O., Neagoe I. (2011). The Relationships Between Biological Activities and Structure of Flavan-3-Ols. Int. J. Mol. Sci..

[18] Fraga C.G., Galleano M., Verstraeten S.V., Oteiza P.I. (2010). Basic biochemical mechanisms behind the health benefits of polyphenols. Mol. Aspects Med..

[19] Spencer J.P.E., Chaudry F., Pannala A.S., Srai S.K., Debnam E., Rice-Evans C. (2000). Decomposition of cocoa procyanidins in the gastric milieu. Biochem. Biophys. Res. Commun..

[20] Bennett R.N., Scalbert A., Rémésy C., Rios L.Y., Williamson G., Lazarus S.A. (2018). Cocoa procyanidins are stable during gastric transit in humans. Am. J. Clin. Nutr..

[21] Hackman R.M., Polagruto J.A., Zhu Q.Y., Sun B., Fujii H., Keen C.L. (2008). Flavanols: Digestion, absorption and bioactivity. Phytochem. Rev..

[22] Kwik-Uribe C., Bektash R.M. (2008). Cocoa flavanols: Measurement, bioavailability and bioactivity. Asia. Pac. J. Clin. Nutr..

[23] Stoupi S., Williamson G., Drynan J.W., Barron D., Clifford M.N. (2010). A comparison of the in vitro biotransformation of (-)-epicatechin and procyanidin B2 by human faecal microbiota. Mol. Nutr. Food Res..

[24] Camps-Bossacoma M., Pérez-Cano F.J., Franch À., Untersmayr E., Castell M. (2017). Effect of a cocoa diet on the small intestine and gut-associated lymphoid tissue composition in an oral sensitization model in rats. J. Nutr. Biochem..

[25] Arola-Arnal A., Muguerza B., Margalef M., Bravo F.I., Iglesias-Carres L., Pons Z. (2017). Flavanol plasma bioavailability is affected by metabolic syndrome in rats. Food Chem..

[26] Holt R.R., Lazarus  S.A., Sullards M.C., Zhu Q.Y., Schramm D.D., Hammerstone J.F., Fraga C.G., Schmitz H.H., Keen C.L. (2018). Procyanidin dimer B2 [epicatechin-(4β-8)-epicatechin] in human plasma after the consumption of a flavanol-rich cocoa. Am. J. Clin. Nutr..

[27] Baba S., Osakabe N., Natsume M., Muto Y., Takizawa T., Terao J. (2001). Absorption and urinary excretion of (-)-epicatechin after administration of different levels of cocoa powder or (-)-epicatechin in rats. J. Agric. Food Chem..

[28] Takizawa T., Baba S., Terao J., Osakabe N., Muto Y., Natsume M. (2018). In Vivo Comparison of the Bioavailability of (+)-Catechin, (−)-Epicatechin and Their Mixture in Orally Administered Rats. J. Nutr..

[29] Ottaviani J.I., Heiss C., Spencer J.P.E., Kelm M., Schroeter H. (2018). Recommending flavanols and procyanidins for cardiovascular health: Revisited. Mol. Aspects Med..

[30] Actis-Goretta L., Lévèques A., Giuffrida F., Romanov-Michailidis F., Viton F., Barron D., Duenas-Paton M., Gonzalez-Manzano S., Santos-Buelga C., Williamson G. (2012). Elucidation of (-)-epicatechin metabolites after ingestion of chocolate by healthy humans. Free Radic Biol. Med..

[31] Aron P.M., Kennedy J.A. (2008). Flavan-3-ols: Nature, occurrence and biological activity. Mol. Nutr. Food Res..

[32] Ohtake Y., Moriichi N., Shoji T., Masumoto S., Akiyama H., Kanda T., Goda Y. (2006). Apple Procyanidin Oligomers Absorption in Rats after Oral Administration: Analysis of Procyanidins in Plasma Using the Porter Method and High-Performance Liquid Chromatography/Tandem Mass Spectrometry. J. Agric Food Chem..

[33] Appeldoorn M.M., Vincken J.-P., Gruppen H., Hollman P.C.H. (2009). Procyanidin Dimers A1, A2, and B2 Are Absorbed without Conjugation or Methylation from the Small Intestine of Rats. J. Nutr..

[34] Llorente-Cortés V., Urpi-Sarda M., Sacanella E., Camino-López S., Vázquez-Agell M., Chiva-Blanch G., Tobias E., Rourae E., Andres-Lacueva C., Lamuela-Raventos R.M. (2011). Cocoa consumption reduces NF-κB activation in peripheral blood mononuclear cells in humans. Nutr. Metab. Cardiovasc. Dis..

[35] Aherne S.A., O ’brien N.M. (2002). Dietary Flavonols: Chemistry, Food Content, and Metabolism chemistry and structure of the flavonoids. Nutrition.

[36] Williamson G., Clifford M.N. (2017). Role of the small intestine, colon and microbiota in determining the metabolic fate of polyphenols. Biochem. Pharmacol..

[37] Verstraeten S.V., Oteiza P.I., Fraga C.G. (2004). Membrane effects of Cocoa procyanidins in liposomes and Jurkat T cells. Biol. Res..

[38] Oteiza P.I., Erlejman A.G., Verstraeten S.V., Keen C.L., Fraga C.G. (2005). Flavonoid-membrane interactions: A protective role of flavonoids at the membrane surface?. Clin. Dev. Immunol..

[39] Verstraeten S.V., Keen C.L., Schmitz H.H., Fraga C.G., Oteiza P.I. (2003). Flavan-3-ols and procyanidins protect liposomes against lipid oxidation and disruption of the bilayer structure. Free Radic Biol. Med..

[40] Repetto M., Semprine J., Boveris A. (2012). Lipid Peroxidation: Chemical Mechanism, Biological Implications and Analytical Determination. InTechOpen.

[41] Perron N.R., Brumaghim J.L. (2009). A review of the antioxidant mechanisms of polyphenol compounds related to iron binding. Cell Biochem. Biophys..

[42] Cherrak S.A., Mokhtari-Soulimane N., Berroukeche F., Bensenane B., Cherbonnel A., Merzouk H., Elhabiri M. (2016). In vitro antioxidant versus metal ion chelating properties of flavonoids: A structure-activity investigation. PLoS ONE.

[43] Loke W.M., Proudfoot J.M., Hodgson J.M., McKinley A.J., Hime N., Magat M., Stocker R., Croft K.D. (2010). Specific dietary polyphenols attenuate atherosclerosis in apolipoprotein e-knockout mice by alleviating inflammation and endothelial dysfunction. Arterioscler. Thromb. Vasc. Biol..

[44] Cheng Y.-C., Sheen J.-M., Hu W.L., Hung Y.-C. (2017). Polyphenols and Oxidative Stress in Atherosclerosis-Related Ischemic Heart Disease and Stroke. Oxid. Med. Cell Longev..

[45] Ali F., Ismail A., Kersten S. (2014). Molecular mechanisms underlying the potential antiobesity-related diseases effect of cocoa polyphenols. Mol. Nutr. Food Res..

[46] Cordero-Herrera I., Martín M.A., Goya L., Ramos S. (2015). Cocoa flavonoids protect hepatic cells against high-glucose-induced oxidative stress: Relevance of MAPKs. Mol. Nutr. Food Res..

[47] Goya L., Martin M.Á., Ramos S. (2016). Antidiabetic actions of cocoa flavanols. Mol. Nutr. Food Res..

[48] Fraga C.G., Oteiza P.I. (2011). Dietary flavonoids: Role of (-)-epicatechin and related procyanidins in cell signaling. Free Radic Biol. Med..

[49] Van Guilder G.P., Hoetzer G.L., Greiner J.J., Stauffer B.L., DeSouza C.A. (2006). Influence of metabolic syndrome on biomarkers of oxidative stress and inflammation in obese adults. Obesity.

[50] Catrysse L., van Loo G. (2017). Inflammation and the Metabolic Syndrome: The Tissue-Specific Functions of NF-κB. Trends. Cell Biol..

[51] Gu Y., Yu S., Lambert J.D. (2014). Dietary cocoa ameliorates obesity-related inflammation in high fat-fed mice. Eur. J. Nutr..

[52] Telle-Hansen V.H., Christensen J.J., Ulven S.M., Holven K.B. (2017). Does dietary fat affect inflammatory markers in overweight and obese individuals?—A review of randomized controlled trials from 2010 to 2016. Genes Nutr..

[53] Schewe T., Steffen Y., Sies H. (2008). How do dietary flavanols improve vascular function? A position paper. Arch. Biochem. Biophys..

[54] Steffen Y., Gruber C., Schewe T., Sies H. (2008). Mono-O-methylated flavanols and other flavonoids as inhibitors of endothelial NADPH oxidase. Arch. Biochem. Biophys..

[55] Rassaf T., Kelm M. (2008). Cocoa flavanols and the nitric oxide-pathway: targeting endothelial dysfunction by dietary intervention. Drug Discov. Today Dis. Mech..

[56] Gómez-Guzmán M., Jiménez R., Sánchez M., Zarzuelo M.J., Galindo P., Quintela A.M., López-Sepulveda R., Romero M., Tamargo J., Vargas F. (2011). Epicatechin lowers blood pressure, restores endothelial function, and decreases oxidative stress and endothelin-1 and NADPH oxidase activity in DOCA-salt hypertension. Free Radic. Biol. Med..

[57] Reiter C.E.N., Kim J.A., Quon M.J. (2010). Green tea polyphenol epigallocatechin gallate reduces endothelin-1 expression and secretion in vascular endothelial cells: Roles for AMP-activated protein kinase, Akt, and FOXO1. Endocrinology.

[58] Yahfoufi N., Alsadi N., Jambi M., Matar C. (2018). The Immunomodulatory and Anti-Inflammatory Role of Polyphenols. Nutrients.

[59] Heiss C., Lauer T., Dejam A., Kleinbongard P., Hamada S., Rassaf T., Matern S., Feelisch M., Kelm M. (2006). Plasma nitroso compounds are decreased in patients with endothelial dysfunction. J. Am. Coll. Cardiol..

[60] Schnorr O., Brossette T., Momma T.Y., Kleinbongard P., Keen C.L., Schroeter H., Sies H. (2008). Cocoa flavanols lower vascular arginase activity in human endothelial cells in vitro and in erythrocytes in vivo. Arch. Biochem. Biophys..

[61] Hajer G.R., Van Haeften T.W., Visseren F.L.J. (2008). Adipose tissue dysfunction in obesity, diabetes, and vascular diseases. Eur. Heart J..

[62] Mackenzie G.G., Delfino J.M., Keen C.L., Fraga C.G., Oteiza P.I. (2009). Dimeric procyanidins are inhibitors of NF-κB-DNA binding. Biochem. Pharmacol..

[63] Fuster J.J., Ouchi N., Gokce N., Walsh K. (2016). Obesity-induced changes in adipose tissue microenvironment and their impact on cardiovascular disease. Circ. Res..

[64] Gonzales A.M., Orlando R.A. (2008). Curcumin and resveratrol inhibit nuclear factor-kappaB-mediated cytokine expression in adipocytes. Nutr. Metab..

[65] Grattagliano I., Palmieri V.O., Portincasa P., Moschetta A., Palasciano G. (2008). Oxidative stress-induced risk factors associated with the metabolic syndrome: a unifying hypothesis. J. Nutr. Biochem..

[66] Steffen Y., Schewe T., Sies H. (2005). Epicatechin protects endothelial cells against oxidized LDL and maintains NO synthase. Biochem. Biophys. Res. Commun..

[67] Steffen Y., Jung T., Klotz L.O., Schewe T., Grune T., Sies H. (2007). Protein modification elicited by oxidized low-density lipoprotein (LDL) in endothelial cells: Protection by (-)-epicatechin. Free Radic Biol. Med..

[68] Ramirez-Sanchez I., Maya L., Ceballos G., Villarreal F. (2010). (-)-Epicatechin activation of endothelial cell endothelial nitric oxide synthase, nitric oxide, and related signaling pathways. Hypertension.

[69] Mendoza-Lorenzo P., Ceballos G., Villarreal F., Varela C.E., Rodriguez A., Romero-Valdovinos M., Mendoza-Loren P., Ramirez-Sanchez I. (2017). Browning effects of (-)-epicatechin on adipocytes and white adipose tissue. Eur. J. Pharmacol..

[70] Jia L., Liu X., Bai Y.Y., Li S.H., Sun K., He C. (2010). Short-term effect of cocoa product consumption on lipid profile: A meta-analysis of randomized controlled trials 1–3. Am. J. Clin. Nutr..

[71] Tokede O.A., Ellison C.R., Pankow J.S., North K.E., Hunt S.C., Kraja A.T., Arnett D.K., Djoussé L. (2012). Chocolate consumption and prevalence of metabolic syndrome in the NHLBI Family Heart Study. e-SPEN J..

[72] Bladé C., Arola L., Salvadó M.J. (2010). Hypolipidemic effects of proanthocyanidins and their underlying biochemical and molecular mechanisms. Mol. Nutr. Food Res..

[73] Rabadan-Chávez G., Reyes-Maldonado E., Quevedo-Corona L., Jaramillo-Flores M.E., Miliar G.A. (2015). Cocoa powder, cocoa extract and epicatechin attenuate hypercaloric diet-induced obesity through enhanced β-oxidation and energy expenditure in white adipose tissue. J. Funct. Foods.

[74] Natsume M., Kanegae M., Yasuda A., Sasaki K., Nagaoka S., Baba S., Natsume M. (2009). Cacao procyanidins reduce plasma cholesterol and increase fecal steroid excretion in rats fed a high-cholesterol diet. BioFactors.

[75] Mellor D.D., Sathyapalan T., Kilpatrick E.S., Beckett S., Atkin S.L. (2010). High-cocoa polyphenol-rich chocolate improves HDL cholesterol in Type 2 diabetes patients. Diabet. Med..

[76] Osakabe N., Yamagishi M. (2009). Procyanidins in Theobroma cacao Reduce Plasma Cholesterol Levels in High Cholesterol-Fed Rats. J. Clin. Biochem. Nutr..

[77] Janevski M., Antonas K.N., Sullivan-Gunn M.J., McGlynn M.A., Lewandowski P.A. (2011). The effect of cocoa supplementation on hepatic steatosis, reactive oxygen species and LFABP in a rat model of NASH. Comp. Hepatol..

[78] Tomaru M., Takano H., Osakabe N., Yasuda A., Inoue K., Yanagisawa R., Ohwatari T., Uematsu H. (2007). Dietary supplementation with cacao liquor proanthocyanidins prevents elevation of blood glucose levels in diabetic obese mice. Nutrition.

[79] Faizul H.A., Nawalyah A.G., Hamid M., Ruzaidi A., Amin I. (2005). The effect of Malaysian cocoa extract on glucose levels and lipid profiles in diabetic rats. J. Ethnopharmacol..

[80] León L., De la Rosa D., Gracia A., Barranco D., Rallo L. (2008). Fatty acid composition of advanced olive. J. Food Sci. Agric..

[81] Grassi D., Lippi C., Desideri G., Necozione S., Ferri C. (2018). Short-term administration of dark chocolate is followed by a significant increase in insulin sensitivity and a decrease in blood pressure in healthy persons. Am. J. Clin. Nutr..

[82] Yamashita Y., Okabe M., Natsume M., Ashida H. (2012). Prevention mechanisms of glucose intolerance and obesity by cacao liquor procyanidin extract in high-fat diet-fed C57BL/6 mice. Arch. Biochem. Biophys..

[83] Gondoin A., Grussu D., Stewart D., McDougall G.J. (2010). White and green tea polyphenols inhibit pancreatic lipase in vitro. Food Res. Int. [Internet]..

[84] Osada K., Suzuki T., Kawakami Y., Senda M., Kasai A., Sami M., Ohta Y., Kanda T., Ikeda M. (2006). Dose-dependent hypocholesterolemic actions of dietary apple polyphenol in rats fed cholesterol. Lipids.

[85] Rabadán-Chávez G.M., Miliar Garcia A., Paniagua Castro N., Escalona Cardoso G., Quevedo-Corona L., Reyes-Maldonado E., Jaramillo Flores M.E. (2016). Modulating the expression of genes associated with hepatic lipid metabolism, lipoperoxidation and inflammation by cocoa, cocoa extract and cocoa flavanols related to hepatic steatosis induced by a hypercaloric diet. Food Res. Int..

[86] Tsukamoto T., Nakata R., Tamura E., Kosuge Y., Kariya A., Katsukawa M., Mishima S., Ito T., Iinuma M., Akao Y. (2010). Vaticanol C, a resveratrol tetramer, activates PPARalpha and PPARbeta/delta in vitro and in vivo. Nutr. Metab..

[87] Jiang H., Zhou X.-Y., Gao D.-M., Zhou N.-J., Xie J., Chen J. (2011). Naringin ameliorates metabolic syndrome by activating AMP-activated protein kinase in mice fed a high-fat diet. Arch. Biochem. Biophys..

[88] Najim N.I., Shah S.A., Kazi A.N., Dharani A.M., Shah S.R., Jangda M.A. (2017). Use of dark chocolate for diabetic patients: a review of the literature and current evidence. J. Commu. Hosp. Intern. Med. Perspect..

[89] Reygaert W. (2017). An Update on the Health Benefits of Green Tea. Beverages.

